# The Broadly Neutralizing DEV_K57 Antibody, Isolated from a Convalescent Patient, in Complex with the SARS-CoV-2 RBD

**DOI:** 10.3390/ijms27219445

**Published:** 2026-10-23

**Authors:** Elina V. Sinitsina, Ilnaz R. Imatdinov, Vitaliy Y. Tuyrin, Andrey V. Shipovalov, Anna V. Zaykovskaya, Irina A. Osinkina, Alexey O. Yanshin, Nikita S. Radchenko, Yury L. Ryzhykau, Vsevolod V. Sudarev, Daria D. Kuklina, Galina S. Onkhonova, Elena K. Ivleva, Alexander P. Agafonov, Anastasia V. Gladysheva

**Affiliations:** 1 State Research Center of Virology and Biotechnology “Vector”, 630559 Kol’tsovo, Russia; 2 Moscow Center for Advanced Studies, 123592 Moscow, Russia

**Keywords:** *Coronaviridae*, SARS-CoV-2, broad neutralization, monoclonal antibodies, hamster model, small-angle X-ray scattering, antigen-antibody complex

## Abstract

The continuous evolution of SARS-CoV-2 necessitates broadly neutralizing antibodies for robust therapeutics. In this study, we isolated 1824 single B cells from a COVID-19 convalescent donor and obtained 91 pairs of nucleotide sequences potentially encoding VH/VL domains. From this library, we generated and evaluated a panel of 38 recombinant monoclonal antibodies. Among this panel, we identified DEV_K57 as a broadly neutralizing human IgG1 antibody targeting the SARS-CoV-2 RBD. DEV_K57 exhibited in vitro neutralization breadth against diverse variants, demonstrating subnanomolar IC_50_ values. This in vitro efficacy was translated in vivo. In a Syrian hamster model of SARS-CoV-2 Delta infection, DEV_K57 significantly reduced viral RNA and infectious titers in both the upper and lower respiratory tracts, conferring a favorable safety profile with minimal histopathological damage. To elucidate its mechanism, we performed comprehensive biophysical and structural analyses. Biolayer interferometry revealed a high picomolar binding affinity to the RBD Delta. Furthermore, size-exclusion chromatography coupled with small-angle X-ray scattering and computational modeling uncovered a unique, stable dimeric architecture of the DEV_K57 Fab-RBD complex in solution, with a binding interface distinct from previously characterized structures. These findings underscore the value of rapid antibody isolation for pandemic preparedness against future emerging biological threats.

## 1. Introduction

Despite the official end of the global health emergency, SARS-CoV-2 (*Coronaviridae* family, *Betacoronavirus* genus, *Betacoronavirus pandemicum* species) continues to circulate and evolve, posing an ongoing threat to public health [1,2,3]. While vaccines have been highly effective in reducing severe disease, the development of specific antiviral therapeutics remains a critical priority [4,5,6]. Among these, virus-neutralizing monoclonal antibodies (mAbs) represent a highly specific and potent class of biologics for both the treatment and prophylaxis of COVID-19 [7,8].

SARS-CoV-2 is an enveloped, positive-sense single-stranded RNA (ssRNA(+)) virus with a spherical morphology and diameter of ~70–160 nm. The viral envelope is studded with club-shaped spike (S) proteins, which give the virus its characteristic crown-like appearance, from which the *Coronaviridae* family derives its name. The virion contains 4 major structural proteins: S glycoprotein, envelope (E) protein, membrane (M) protein, and nucleocapsid (N) protein [9,10,11]. The S glycoprotein consists of 2 functional subunits, S1 and S2, and is arranged as a homotrimer on the surface of the viral membrane [12,13,14]. The primary target for neutralizing mAbs is the S glycoprotein of SARS-CoV-2, specifically the receptor-binding domain (RBD) within the S1 subunit. The RBD mediates viral entry by binding to the host cell receptor angiotensin-converting enzyme 2 (ACE2). By blocking this interaction or inducing steric hindrance, neutralizing mAbs effectively prevents viral infection [15,16,17,18]. Initially, several mAb therapies and cocktails received emergency use authorization and demonstrated significant clinical efficacy in reducing hospitalization and mortality. These include the REGN-COV2 cocktail containing Casirivimab and Imdevimab (Regeneron Pharmaceuticals, Tarrytown, NY, USA) and the combination of Bamlanivimab and Etesevimab (Eli Lilly and Company, Indianapolis, IN, USA) [19,20,21,22,23].

However, the continuous evolution of SARS-CoV-2 has presented a major challenge to mAb-based therapies. The accumulation of mutations, particularly within the RBD of variants of concern, has led to significant antigenic drift [24]. Consequently, most early-generation mAbs have lost their neutralizing potency against circulating variants and were subsequently withdrawn from clinical use. A representative example is GamCoviMab (National Research Center for Epidemiology and Microbiology named after Honorary Academician N.F. Gamaleya, Moscow, Russia), a therapeutic preparation containing the P2C5 antibody, which successfully completed phase I clinical trials. However, following the emergence and widespread circulation of the Delta variant carrying the L452R mutation and the Omicron XBB.1 variant carrying the F490S mutation within the RBD, a marked reduction in the neutralizing activity of this clone was observed [24,25]. In response to this challenge, the same research group later reported the development of a novel mAb, 1p1B10, which exhibits broad neutralizing activity against both previously circulating and novel SARS-CoV-2 variants. Notably, the RBD-1p1B10 interaction was shown to remain largely unaffected by the accumulated RBD mutations and to substantially overlap with the RBD-ACE2 binding interface [26]. Despite such encouraging advances, the rapid immune evasion of SARS-CoV-2 underscores the urgent need to not only identify and characterize novel broadly neutralizing antibodies, but also to elucidate the structural basis of their broad reactivity. Such structural insights are essential for the rational design of therapeutics that can maintain efficacy against diverse and highly mutated viruses [27].

In the present study, we report the isolation of a novel virus-neutralizing antibody, DEV_K57, from a convalescent patient, which demonstrates potent broadly neutralizing activity against multiple SARS-CoV-2 variants. To elucidate the molecular basis of this broad reactivity and understand its interaction with the RBD, we report the solution structure of the DEV_K57 Fab-RBD complex. Using size-exclusion chromatography coupled with small-angle X-ray scattering (SEC-SAXS), we characterized the overall structural organization and conformational dynamics of the complex under near-native conditions.

## 2. Results

The mAbs described in this study were isolated from a single convalescent donor following infection with the SARS-CoV-2 Delta variant in 2023. Initial serum samples from this donor exhibited high virus-neutralizing activity against multiple SARS-CoV-2 variants, prompting comprehensive characterization of the antibody repertoire.

### 2.1. Generation of Recombinant Monoclonal Antibodies from Convalescent B Cells

**Figure 1:**
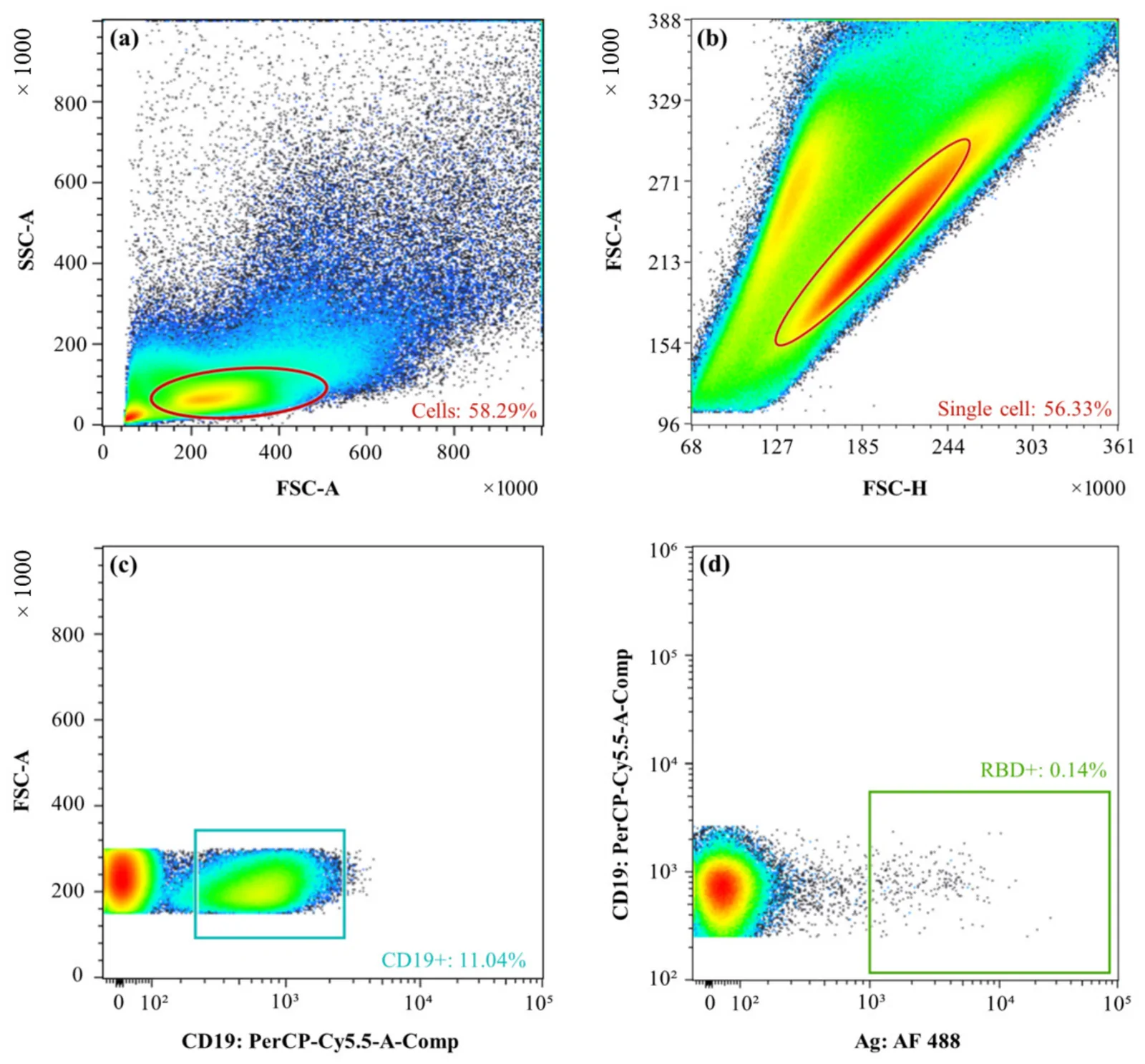
Gating strategy for the single-cell sorting of SARS-CoV-2 RBD-specific B-lymphocytes from a convalescent donor (CD19^+^RBD^+^). (**a**) Lymphocyte population gated by forward scatter area (FSC-A) and side scatter area (SSC-A). (**b**) Singlet selection using FSC-A versus forward scatter height (FSC-H). (**c**) Viable singlet CD19^+^ B-lymphocytes gated based on PerCP-Cy5.5 fluorescence intensity. (**d**) Final sorting gate for viable, single, CD19^+^, RBD-specific (Alexa Fluor 488^+^) B lymphocytes.

We isolated 1824 single B cells and subjected them to VH/VL-specific PCR amplification (Figure 1). As a result, we obtained 91 pairs of nucleotide sequences potentially encoding VH/VL domains. From the formed cDNA library, 40 pairs were selected for further analysis. The VH genes originated from the germlines IGHV1, IGHV2, IGHV3, IGHV4, and IGHV5, with IGHV3 representing the highest proportion (53.6%). The VL sequences included 21 kappa (κ) and 19 lambda (λ) light chains, with the predominant germlines being IGKV1 (57.1%) and IGLV3 (38.8%), respectively (Figure S1).

As a result, 38 recombinant plasmids encoding the variable and constant domains of immunoglobulin heavy and light chains were obtained, along with stable CHO-S producer cell lines.

ELISA analysis revealed that the recombinant mAbs exhibited varying specificity against SARS-CoV-2 Delta and Omicron BA.5.1 variants (Table S1). Specifically, 18 antibody variants showed specific reactivity to the Delta variant, 28 reacted with the Omicron variant, and 14 demonstrated cross-reactivity to both Delta and Omicron. Among the 38 analyzed mAbs, 5 exhibited no specific reactivity to the tested SARS-CoV-2 variants in the ELISA assay, yielding OD450 values below the defined positivity thresholds.

All 38 mAbs were analyzed for their virus-neutralizing activity against SARS-CoV-2 Delta and Omicron BA.5.1 variants (Table 1 and Table S1). Six recombinant mAbs possessed virus-neutralizing activity against both SARS-CoV-2 Delta and Omicron BA.5.1 variants (DEV_K57, DEV_K51, DEV_K61, DEV_K55, DEV_L45, and DEV_L33). Among the recombinant mAbs, 12 exhibited neutralizing activity against only 1 variant (8 against Delta and 4 against Omicron), while 20 did not demonstrate virus-neutralizing activity at the tested concentrations.

By comparing the results of ELISA and virus-neutralization, four mAbs (DEV_K57, DEV_K51, DEV_K61, and DEV_K55) were identified that specifically interacted with both SARS-CoV-2 variants and demonstrated virus-neutralizing activity against them (Table S2).

### 2.2. Neutralizing Activity of DEV_K57 Against SARS-CoV-2 Variants

DEV_K57 demonstrated the most promising neutralizing activity. Therefore, we additionally evaluated its neutralizing potency against a broad panel of SARS-CoV-2 variants available by the end of 2023, including the ancestral Wuhan strain, Alpha, Beta, Gamma, Delta, and various Omicron lineages (Table 2). Analysis shows that DEV_K57 exhibits potent in vitro neutralizing activity against the majority of SARS-CoV-2 variants. Notably, DEV_K57 neutralized the ancestral Wuhan, Alpha, Beta, Gamma, Delta, Omicron BA.1, BA.2, and BN.1.3 with high efficiency (from 0.005 IC_50_ to > 0.0005 IC_50_). The antibody also retained activity against later Omicron subvariants, including BA.5.2 and BQ.1.1 (5 IC_50_), BA.5.1 (7.81 IC_50_), CL.1.2 (15.63 IC_50_), CH.1.1 (50 IC_50_), XBB.1.16 (125 IC_50_), XBB.1.5 (250 IC_50_), and XBB.3 (500 IC_50_). However, neutralizing activity was significantly reduced against EG.5.1 and JN.1 (>500 IC_50_), indicating partial immune evasion by this highly mutated variant.

### 2.3. In Vivo Efficacy of DEV_K57 in the SARS-CoV-2 Delta-Infected Golden Syrian Hamster Model

**Figure 2:**
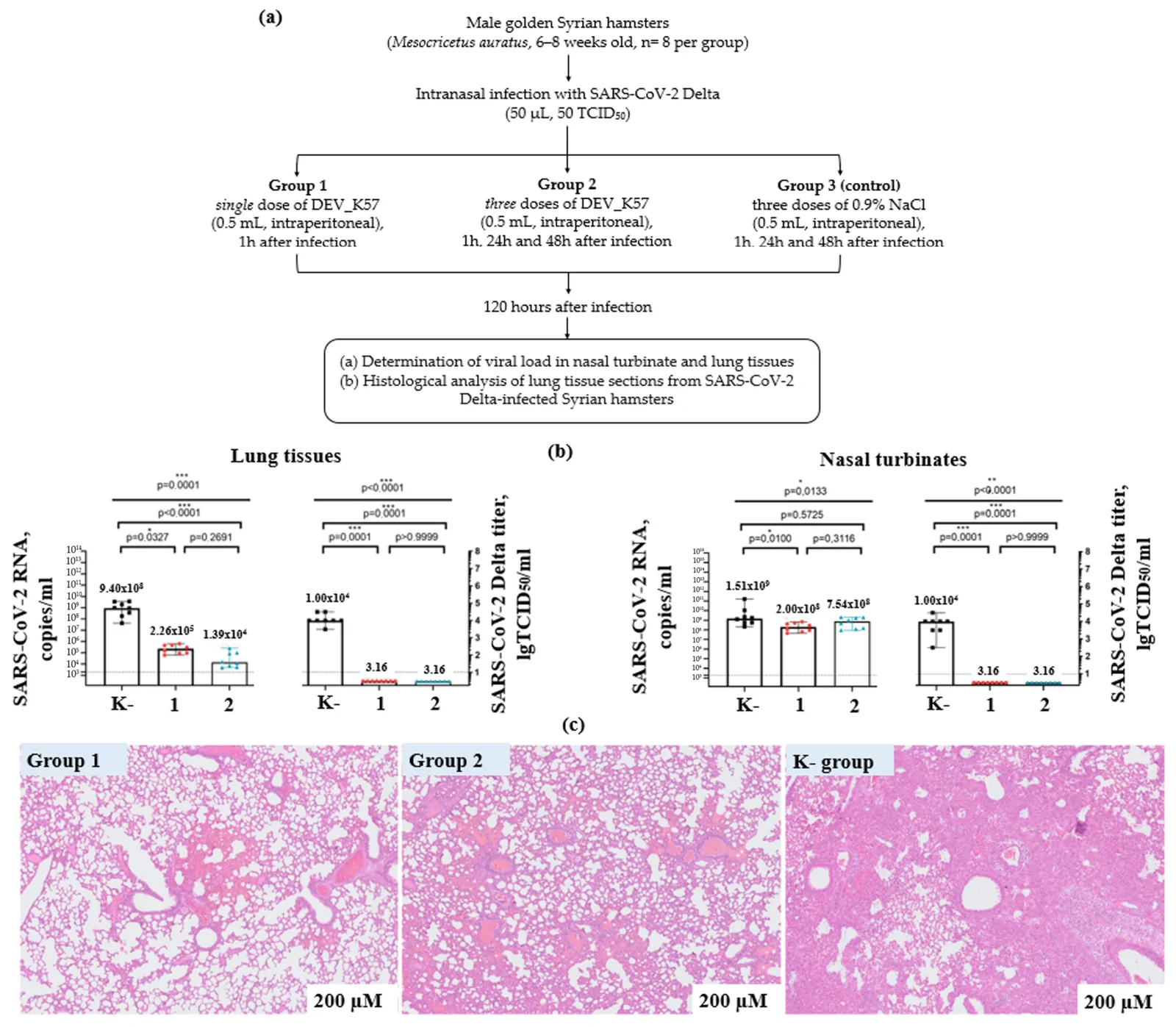
Efficacy of DEV_K57 in the SARS-CoV-2 Delta-infected golden Syrian hamster model. (**a**) Schematic representation of the in vivo experimental design. (**b**) Viral load in the lung tissues (left 2 graphs) and nasal turbinate (right 2 graphs) of Syrian hamsters at 120 h post-intranasal infection. The left y-axis indicates viral RNA concentration in 10% tissue homogenates, and the right y-axis shows infectious virus titers in Vero E6 cells. Each dot represents an individual value, bar tops indicate group medians, and vertical lines represent 95% confidence intervals. Horizontal dotted lines denote threshold values for SARS-CoV-2 RNA (≤10^3.3^ copies/mL) and infectious titers (≤1 log_10_TCID_50_/mL). Statistical differences were evaluated using the Kruskal-Wallis test (*p*-values indicated above horizontal lines). Pairwise group comparisons were performed using Dunn’s post-hoc test (*p*-values indicated above brackets). Significance levels: ns (not significant, *p* ≥ 0.05), * *p* < 0.05, ** *p* < 0.01, *** *p* < 0.001. (**c**) Representative histological sections of lung tissues from SARS-CoV-2 Delta-infected Syrian hamsters. Group 1—single-dose mAb administration group; Group 2—triple-dose mAb administration group; K−—control group.

The efficacy of DEV_K57 was evaluated in a Syrian hamster model of SARS-CoV-2 Delta infection (Figure 2). All treatment groups showed a reduction in infectious viral titers in nasal turbinate and lung tissue samples. Intergroup comparisons using the Kruskal-Wallis test revealed significant heterogeneity (*p* < 0.0001). Dunn’s test demonstrated statistically significant differences in infectious titers in nasal and lung tissues in both the single-dose group (*p* = 0.0001) and the triple-dose group (*p* = 0.0001) compared to the control group.

A decrease in SARS-CoV-2 viral RNA copies was also observed in nasal turbinate and lung tissue samples across all treatment groups (Figure 2b). Intergroup comparisons using the Kruskal-Wallis test revealed significant heterogeneity for viral RNA concentrations in lung tissues (*p* < 0.0001) and nasal turbinate tissues (*p* = 0.0133). Dunn’s test showed statistically significant differences in viral RNA concentrations in lung tissues in the single-dose group (*p* = 0.0327) and the triple-dose group (*p* < 0.0001) compared to the control group. A significant difference in viral RNA concentrations in nasal turbinate tissues was observed only in the single-dose group (*p* = 0.0100).

Single and triple administration of DEV_K57 in SARS-CoV-2-infected Syrian hamsters did not cause severe histopathological changes (Figure 2c). All animals in the single-dose group exhibited similar mild pathological findings, including small focal haemorrhages and minor areas of atelectasis with low intensity. Histological examination of lung tissues from the triple-dose group revealed that 2 animals showed larger areas of atelectasis with greater intensity compared to the single-dose group. Haemorrhages were also more pronounced in these animals. The remaining 6 animals in this group showed lung histology consistent with normal findings. No inflammatory cell infiltration was detected in the experimental groups. Blood vessels and bronchioles appeared normal.

### 2.4. Binding Kinetics of DEV_K57 with SARS-CoV-2 RBD Delta

**Figure 3:**
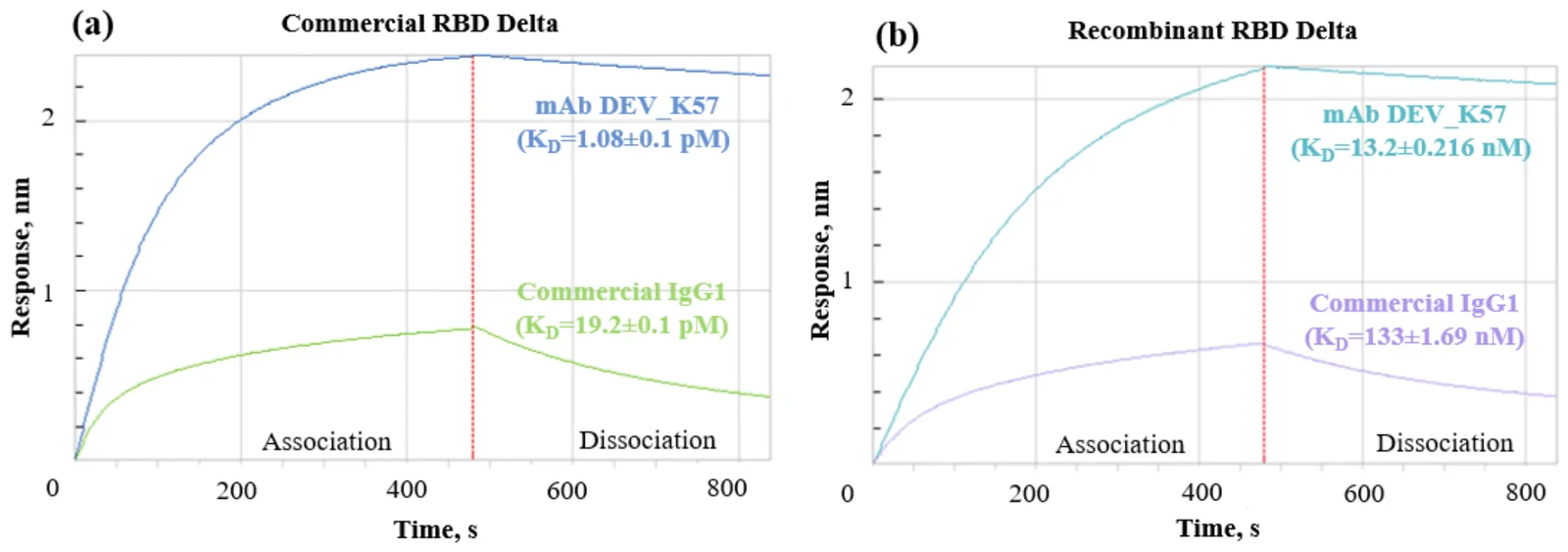
Comparative binding analysis of DEV_K57 (blue and cyan) and commercial IgG1 (green and purple) to SARS-CoV-2 RBD Delta. (**a**) Binding kinetics with commercial SARS-CoV-2 RBD Delta. (**b**) Binding kinetics with in-house recombinant SARS-CoV-2 RBD Delta produced in this study. The red dashed line separates the association and dissociation stages.

To elucidate the molecular basis of the DEV_K57 neutralizing activity, we evaluated its binding kinetics to the commercial SARS-CoV-2 RBD Delta (AFG10876, R&D Systems, Minneapolis, MN, USA) and our recombinant SARS-CoV-2 RBD Delta. Bio-layer interferometry (BLI) analysis revealed an exceptionally high binding affinity, with a dissociation constant (K_D_) = 1.08 ± 0.1 pM and K_D_ = 13.2 ± 0.216 nM, respectively (Figure 3). To benchmark the binding affinity of the newly isolated DEV_K57, we also compared its interaction with the SARS-CoV-2 RBD Delta to that of a commercial IgG1 mAb. DEV_K57 demonstrated a higher affinity than the commercial IgG1 (K_D_ = 19.2 ± 0.1 pM and K_D_ = 133 ± 1.69 nM). This picomolar affinity provides a thermodynamic basis for the antibody’s ability to effectively outcompete the cellular ACE2 receptor.

### 2.5. SEC-SAXS Study of DEV_K57 Fab and SARS-CoV-2 RBD Delta

To establish a structural baseline for the DEV_K57 Fab and SARS-CoV-2 RBD Delta complex, we determined the experimental solution-state conformation of its recombinant complex. SEC-SAXS was specifically employed to isolate the monodisperse fraction for scattering measurements.

**Figure 4:**
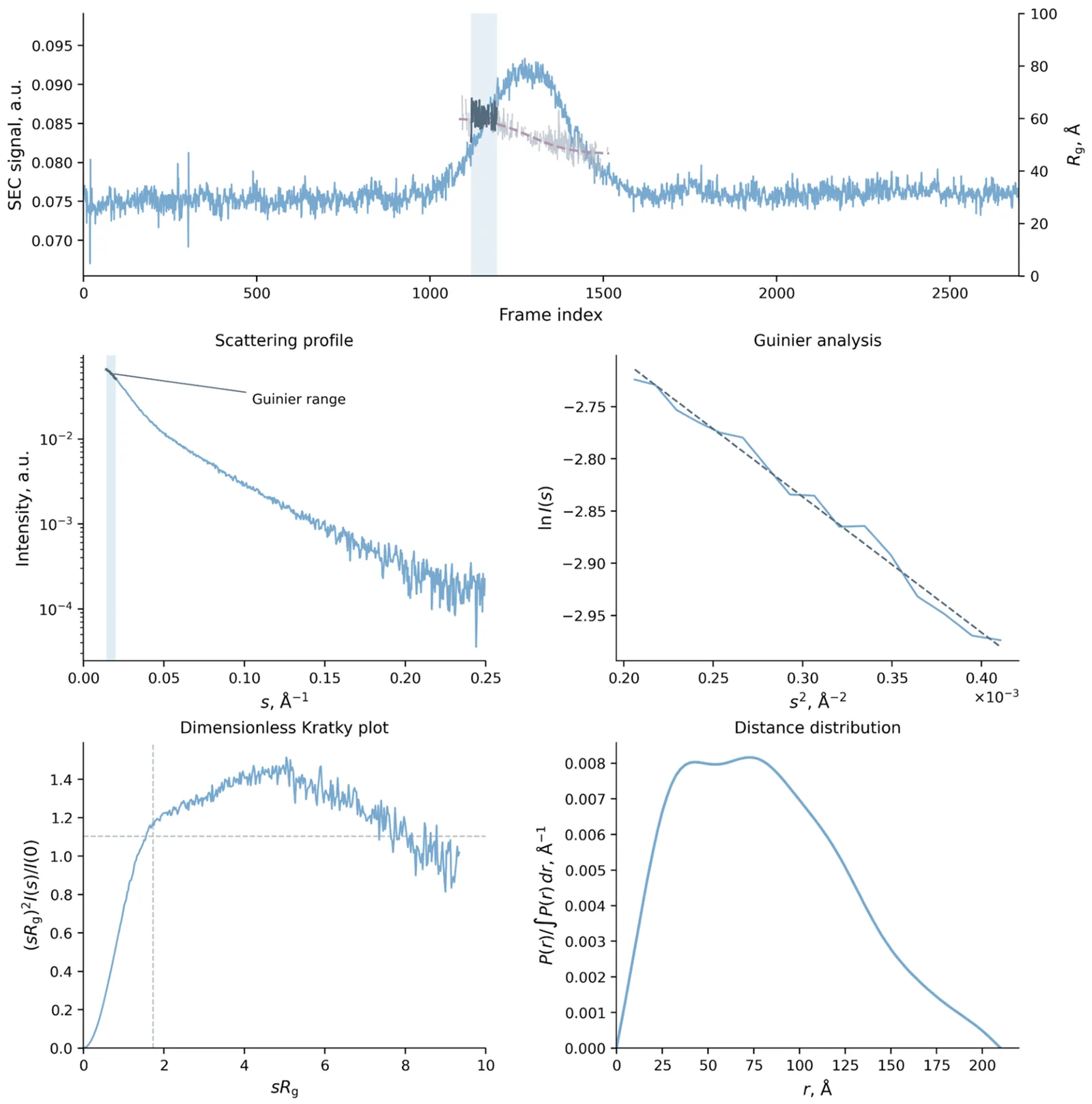
SEC-SAXS analysis of the DEV_K57 Fab-SARS-CoV-2 RBD Delta complex. The top row shows the SEC-SAXS elution profile (light blue) and frame-by-frame Rg values (gray) for frames 1084–1517. The purple dashed line represents the Boltzmann sigmoid function approximation. The vertical blue region marks frames 1119–1209, for which Rg values are shown in dark blue. The middle row shows the buffer-subtracted scattering profile I(s) (**left**), with the shaded interval indicating the Guinier fitting range, and the corresponding Guinier plot (**right**). The bottom row shows the dimensionless Kratky plot (**left**), where dashed lines indicate reference values for a globular particle and the normalized P(r) (**right**).

A total of 2700 SAXS frames were collected continuously during sample elution (Figure 4). The SEC-SAXS data were evaluated across the elution profile. To confirm the homogeneity of the sample, the ratio of ultraviolet absorbance to differential refractive index (UV/dRI) was also monitored (Figure S2). Consequently, the interval spanning frames 1119–1209 was selected for frame-wise Rg analysis (Figure 4).

Guinier analysis of the processed scattering curve yielded Rg = 59.4 Å and I(0) = 0.061 a.u., with a reduced χ^2^ of 0.99. The selected sRg interval of 0.7–1.30 showed a linear dependence of lnI(s) on s^2^, supporting the applicability of the Guinier approximation within the selected low-q range. Real-space analysis using GNOM yielded Rg = 62.4 Å and Dmax = 205 Å. The reciprocal-space Rg reported by GNOM was in close agreement with the real-space value. The resulting Rg/Dmax ratio was approximately 0.3. The dimensionless Kratky profile exhibited a broad maximum at sRg ≈ 4.4, with a normalized height of ~1.43, markedly shifted from the canonical maximum expected for a compact globular particle (Figure 4).

The molecular weight (MW) estimated using various independent computational approaches (Bayesian inference, Qp, Volume of Correlation, MoW) yielded a consistent average of approximately 155–184 kDa. Notably, the Bayesian estimate provided a MW of 169.6 [151.4–176.6] kDa, which is nearly twice the expected molecular weight of a monomeric Fab-RBD complex (~80 kDa). This discrepancy strongly suggests that the SARS-CoV-2 RBD forms a dimer in solution.

**Figure 5:**
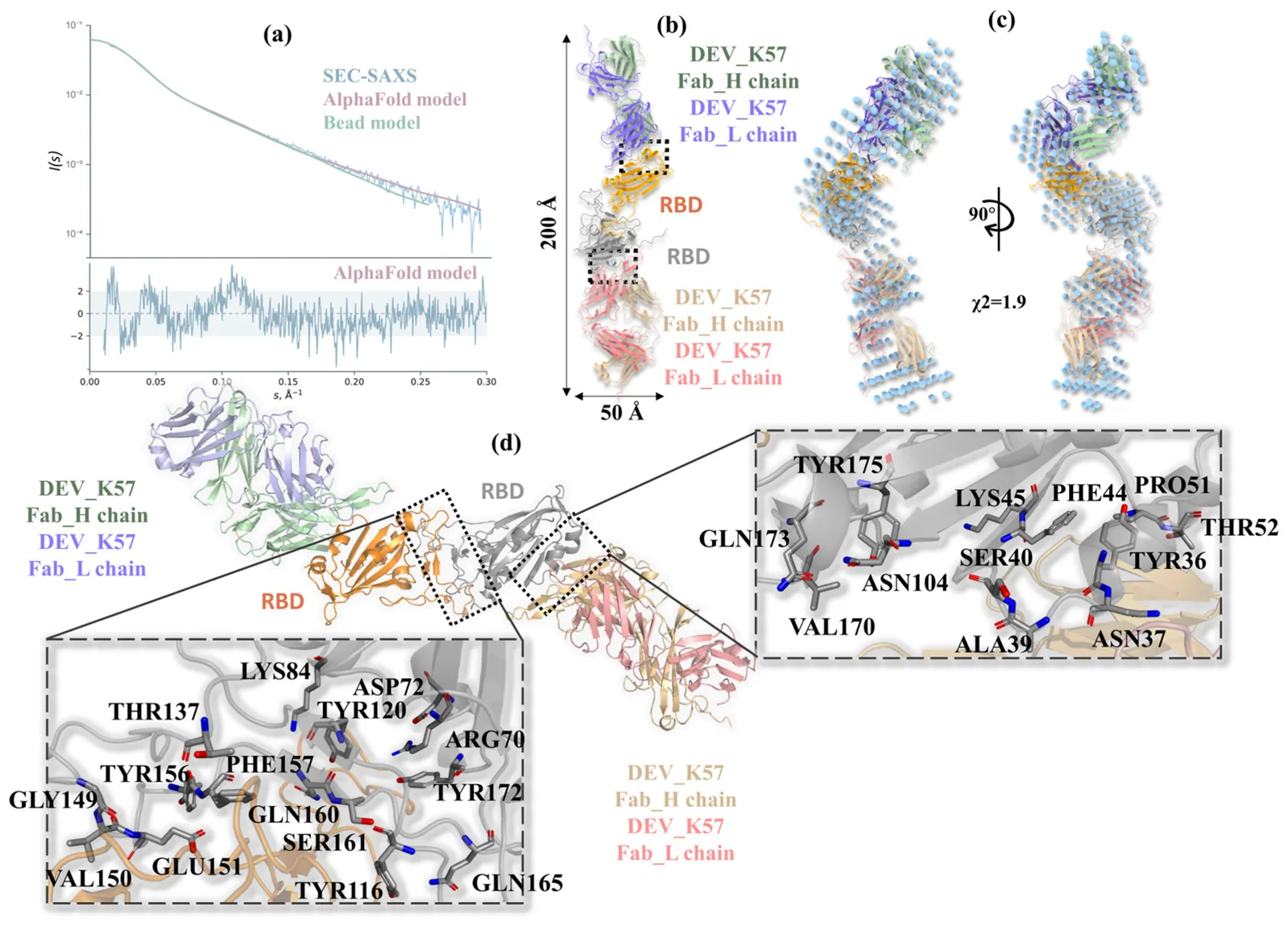
Atom model reconstruction of the DEV_K57 Fab-SARS-CoV-2 RBD Delta complex. (**a**) Comparison of the experimental SAXS scattering curve (blue) with the theoretical scattering profiles calculated from the AlphaFold-predicted model (pink) and the ab initio dummy atom model (green). (**b**) AlphaFold-predicted structure of the dimeric DEV_K57 Fab-SARS-CoV-2 RBD Delta complex. (**c**) Superposition of the ab initio dummy atom envelope (blue dots) and the AlphaFold-predicted dimeric model (colored cartoon representation). (**d**) Detailed views of the RBD-RBD and RBD-DEV_K57 Fab interfaces within the dimeric assembly. All amino acid residues displayed in these detailed views belong to the RBD and are colored gray. The specific amino acid residues of DEV_K57 involved in the interaction are presented in Figure S3.

To visualize the overall shape of the complex, we generated an ab initio dummy atom model using DAMMIF (Figure 5a,c). The reconstructed envelope revealed a dimeric assembly in which 2 SARS-CoV-2 RBD molecules are bridged by 2 DEV_K57 Fab fragments, with each RBD monomer interacting with one Fab (Figure 5b–d). The dimeric arrangement is consistent with the elongated P(r) profile and the elevated MW estimate, providing direct structural evidence for the solution-state dimerization of the complex. We obtained χ^2^ = 1.9 between the experimental scattering curve and the theoretical profile calculated from the AlphaFold model of the RBD-DEV_K57 Fab dimer (Figure 5a,c). The observed discrepancies are likely due to the fact that the recombinant RBD used in this study was produced in HEK293 cells, ensuring native-like glycosylation, while the current AlphaFold model does not explicitly account for these surface glycans. Furthermore, the presence of a hydration shell also influences the scattering profile.

**Figure 6:**
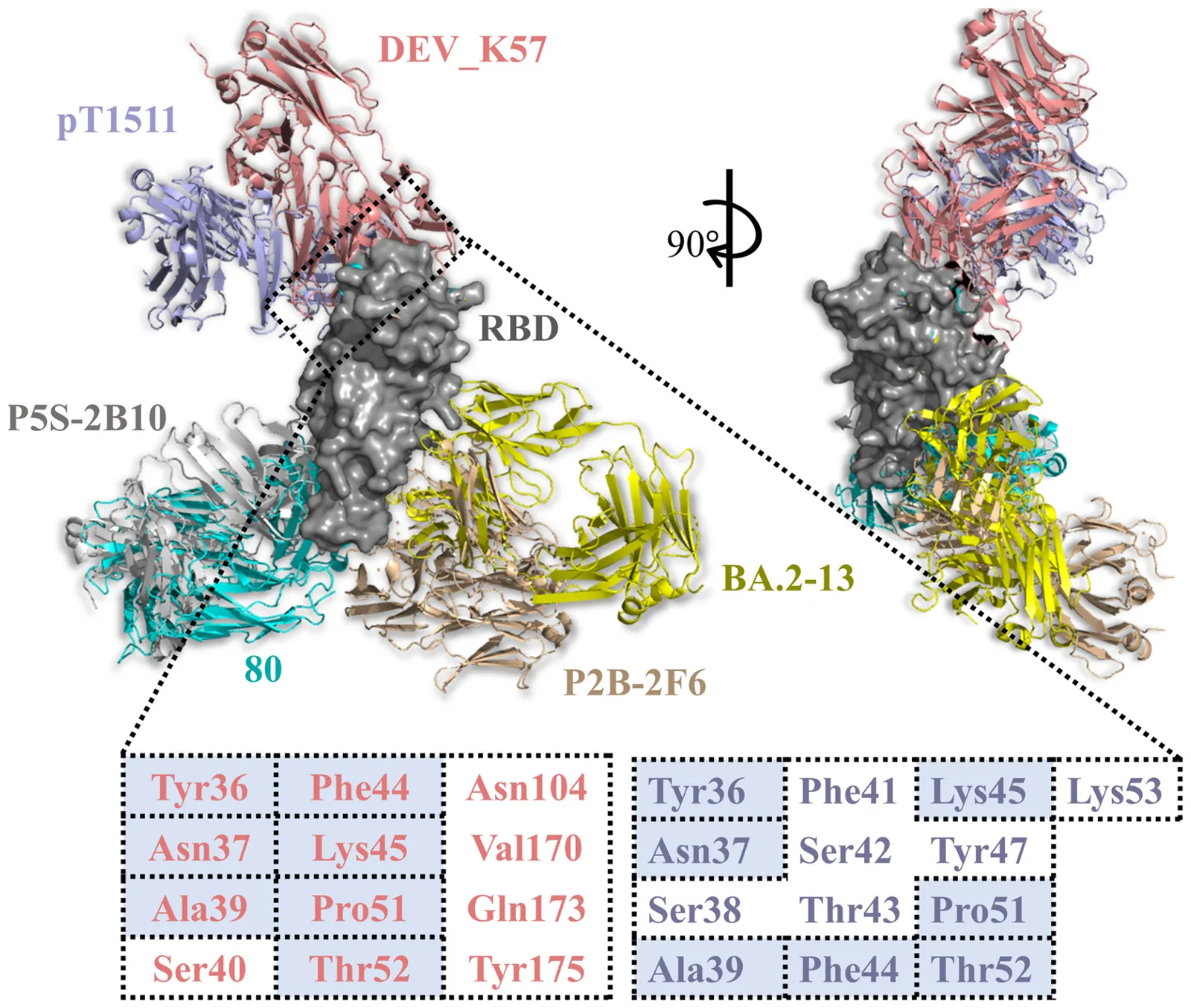
Structural superposition of SARS-CoV-2 RBD-mAb complexes. The complexes shown are DEV_K57 (pink), pT1511 (blue; PDB ID: 8BG1), P5S-2B10 (gray; PDB ID: 7XCS), 80 (cyan; PDB ID: 8DNN), P2B-2F6 (brown; PDB ID: 8DCC), and BA.2-13 (yellow; PDB ID: 8C3V). The SARS-CoV-2 RBD is depicted as a gray molecular surface, while the mAbs are shown in ribbon representation. The lower panel provides a detailed view of the interaction interfaces between the RBD and the DEV_K57 or pT1511 antibodies. The color of the amino acid residues corresponds to the respective antibody, with residues shared by both DEV_K57 and pT1511 highlighted in light blue.

Subsequently, we employed FoldSeek to search for structurally similar RBD-mAb complexes in the PDB. Five homologous structures (PDB IDs: 8BG1, 7XSC, 8DNN, 8DCC, and 8C3V) were selected based on the highest TM-scores (0.78–0.89; Table S3, Figure S4). Analysis revealed that all selected mAbs recognize distinct epitopes on the SARS-CoV-2 RBD surface, which accounts for the differences in their activities. The most structurally similar binding interface to DEV_K57 was found for the pT1511 antibody (PDB ID: 8BG1). Seven RBD amino acid residues were common to the interaction with both DEV_K57 and pT1511 antibodies (Figure 6).

## 3. Discussion

In this study, we generated and evaluated a panel of 38 recombinant mAbs isolated from a COVID-19 convalescent donor. Among this panel, we characterized DEV_K57 as a broadly neutralizing antibody.

DEV_K57 is a human IgG1 monoclonal antibody targeting the RBD of the SARS-CoV-2 spike protein. Notably, DEV_K57 exhibits a broad neutralization spectrum against diverse SARS-CoV-2 variants, including late-stage evolutionary lineages of the Omicron family [28,29]. In vitro assays demonstrated that DEV_K57 potently neutralizes the ancestral Wuhan strain, alongside the Alpha, Beta, Gamma, Delta, Omicron BA.1, BA.2, and BN.1.3 variants. Furthermore, the antibody retained measurable neutralizing activity against more recent subvariants, including BA.5.2, BQ.1.1, BA.5.1, CL.1.2, CH.1.1, XBB.1.16, XBB.1.5, and XBB.3. However, a substantial reduction in neutralizing potency is observed against the highly mutated EG.5.1 and JN.1 lineages, a finding that is fully consistent with recent deep mutational scanning data mapping the antibody escape landscape of contemporary viral lineages [30].

Comparatively, DEV_K57 is distinguished by its exceptional breadth of neutralization. Many previously characterized RBD-targeting antibodies, despite their high efficacy against early strains, have completely lost neutralizing activity against variants such as BA.5, XBB.1.5, BA.2.86, JN.1, and KP.3 [28,31]. Specifically, antibodies such as S2E12, ZCB11, OMI-42, LY-CoV1404, COV2-2130, VIR-7831, and BRII-196 exhibit a marked reduction or complete loss of activity against XBB.1.5 and subsequent variants [28,29].

Furthermore, DEV_K57 surpasses the neutralization breadth of antibodies isolated exclusively following Omicron infection (e.g., 1332E5 and 1332D4). As demonstrated in recent studies, antibodies induced by Omicron infection frequently exhibit pan-Omicron specificity but fail to bind the ancestral Wuhan-1 strain (e.g., 1332E5). Alternatively, they may neutralize KP.3 but are evaded by variants harboring the H445R mutation, such as XFG, as observed with 1332D4 [32]. In contrast, DEV_K57 successfully neutralizes both the ancestral strain and a broad spectrum of Omicron lineages, exhibiting only a gradual and predictable decline in potency as the mutational load within the RBD increases.

While DEV_K57 demonstrates remarkable neutralization breadth across the evolutionary landscape of SARS-CoV-2, the Delta variant was specifically selected as the primary model for our in vivo efficacy and detailed biophysical characterizations.

Crucially, the in vitro neutralizing potency of DEV_K57 translates into robust therapeutic efficacy in vivo. In a golden Syrian hamster model of SARS-CoV-2 Delta infection, both single and triple intraperitoneal administrations of DEV_K57 significantly reduced infectious viral titers and viral RNA loads in both the nasal turbinates and lung tissues compared to the control groups. Furthermore, histopathological evaluation revealed no severe pathological alterations. Treated animals exhibited only mild, focal pathological changes without the severe inflammatory cell infiltration typically associated with SARS-CoV-2 infection. This ability to effectively suppress viral replication in both the upper and lower respiratory tracts, coupled with minimal tissue damage, strongly underscores the translational potential of DEV_K57 as a therapeutic agent.

This in vivo efficacy is fundamentally underpinned by the high binding affinity of DEV_K57 to the SARS-CoV-2 RBD Delta. BLI analysis revealed that DEV_K57 binds to the RBD Delta with a picomolar dissociation constant, which is approximately an order of magnitude higher than that of a commercial reference IgG1. Such robust thermodynamic binding likely allows the antibody to maintain strong interactions with the RBD even when minor structural fluctuations or point mutations occur in highly evolved variants.

SEC-SAXS revealed a dimeric architecture of the DEV_K57 Fab-RBD complex in solution. The experimental molecular weight was approximately twice the theoretical mass of a monomeric complex, indicating that the complex forms a stable dimer. While the isolated RBD is known to possess an intrinsic propensity to dimerize in the absence of the full spike protein, the formation of a stable Fab-RBD dimer suggests that DEV_K57 binding either preserves or significantly stabilizes this RBD-RBD dimeric interface.

Further structural insights into the binding mechanism were gained through computational modeling and comparative structural analysis. We identified 5 homologous RBD-mAb complexes in the PDB with high structural similarity [33,34,35,36]. Notably, the most structurally similar binding interface was observed with the broadly neutralizing antibody pT1511 (PDB ID: 8BG1), with which DEV_K57 shares seven key contact residues within the RBD. These residues represent candidate contact sites for DEV_K57. However, given that a publication detailing the functional characterization of pT1511 is not yet available, we cannot directly extrapolate this structural similarity to functional equivalence.

Our comparative analysis highlights a significant divergence in the epitopes targeted by these homologous antibodies. This epitope divergence is a primary determinant of the varying neutralization profiles and susceptibilities to viral escape mutations observed across different mAbs [37]. Antibodies targeting the highly mutable receptor-binding motif (RBM) are rapidly evaded by contemporary variants. In contrast, mAbs targeting conserved peripheral or cryptic epitopes retain broader neutralization [38].

This study has some limitations. First, the isolation and initial characterization of DEV_K57 were conducted using viral variants circulating up to late 2023. Given the relentless evolutionary trajectory of SARS-CoV-2, continuous surveillance and validation of DEV_K57 against newly emerging variants are essential to confirm its therapeutic viability. Second, while our SEC-SAXS analysis and computational modeling provide robust low-resolution insights into the dimeric architecture and general epitope classification of the DEV_K57-RBD complex, they cannot resolve atomic-level details. Future high-resolution structural studies, particularly X-ray crystallography or cryo-electron microscopy, will be critical to precisely map the paratope–epitope interface. Further work will involve structural optimization of the DEV_K57 paratopes to broaden its neutralizing activity using methods of structural biology and genetic engineering. As part of these studies, DEV_K57 will be tested against later lineages circulating in 2024–2026.

Despite these limitations, our study shows that DEV_K57 is a highly effective, naturally produced human antibody. It achieves broad neutralization and picomolar affinity without artificial laboratory modifications. As the virus evolves, antibodies like DEV_K57 will be valuable tools for creating next-generation treatments against the current and future variants.

## 4. Materials and Methods

### 4.1. Ethical Statement

The collection and use of donor blood were approved by the Ethics Committee (IRB 00001360) of the State Research Center of Virology and Biotechnology “Vector” (Protocol №7 dated 18 April 2023). All in vivo experimental procedures were reviewed and approved by the Bioethics Committee of the State Research Center of Virology and Biotechnology “Vector” (Application number SRC VB VECTOR/14-12.2023, approved by protocol №11 dated 15 December 2023).

### 4.2. Isolation of SARS-CoV-2 RBD-Specific Memory B Cells

B cells were isolated from 45 mL of peripheral blood from a COVID-19 convalescent donor by Ficoll gradient medium centrifugation. Freshly isolated peripheral blood mononuclear cells were incubated with recombinant SARS-CoV-2 RBD Delta in PBS supplemented with 2% fetal bovine serum and an Fc receptor blocking reagent for 60 min at 4 °C, followed by 2 washes. Cells were then stained with anti-His Tag Alexa Fluor 488 (clone J099B12; BioLegend, San Diego, CA, USA), anti-Human CD19 PerCP-Cy5.5 (clone HIB19; BD Pharmingen, San Diego, CA, USA), and Fixable Viability Stain 620 (FVS620; BD Biosciences, San Jose, CA, USA) for 60 min at 4 °C. After 2 additional washes, the cell suspension was filtered through a 70-µm cell strainer. Single-cell sorting was performed using a Sony SH800 (Sony Biotechnology, Tokyo, Japan). The gating strategy included the sequential selection of lymphocytes, singlets, live cells, CD19+ B cells, and finally, RBD-specific B cells. Cells were deposited directly into 96-well PCR plates containing reverse transcription RNAscribe RT Kit (Biolabmix, Novosibirsk, Russia), a 5’ template-switching oligonucleotide (5’-TSO) adapter, and gene-specific primers complementary to the constant domain sequences of immunoglobulin heavy and light chains (R-hIGH_1, R-hIGK_1, and R-hIGL_1; Table 3). Reverse transcription was performed according to the manufacturer’s instructions.

### 4.3. Cloning of Genes Encoding Antibody Variable Domains and Antibody Production

Following reverse transcription, a two-step semi-nested PCR was performed using Q5 High-Fidelity DNA Polymerase (New England Biolabs, Ipswich, MA, USA). The first PCR round used the reverse transcription reaction as a template, with the external 5’ F-iRACE-primer_C (complementary to the 5’-TSO) and a mixture of internal gene-specific primers complementary to the constant domain sequences. The second PCR round amplified the nucleotide sequences encoding the variable domains of heavy and light chains using 1 µL of the first PCR product as a template and specific primers (R-hIGH_3, R-hIGK_3, R-hIGL_3, and F-iL_B; Table 3).

PCR products were separated on a 2% agarose gel, and bands corresponding to VH-CH (500–650 bp) and VL-CL (450–600 bp) were excised and extracted using the Cleanup Standard kit (Eurogen, Moscow, Russia). The VL-CL and VH-CH fragments were sequentially cloned into the SBW7G_HIGH integration vector at EcoRI/XhoI and MluI/NheI restriction sites, respectively (Figure S5a). Each cloning step was confirmed by Sanger sequencing using the BigDye Terminator v3.1 Cycle Sequencing Kit (Thermo Fisher Scientific, Waltham, MA, USA) on an ABI 3500/3500xl Genetic Analyzer (Applied Biosystems, Carlsbad, CA, USA). Paired IgG heavy chain and light chain plasmids were co-transfected into CHO-S cells (Thermo Fisher Scientific, Waltham, MA, USA), and the cells were incubated at 37 °C in a humidified 5% CO_2_ atmosphere for 15 days.

### 4.4. Purification of Recombinant Antibodies

Recombinant monoclonal antibodies were purified from cell culture supernatants by affinity chromatography using WorkBeads Protein A resin (Bio-Works Technologies, Uppsala, Sweden) on an ÄKTA Pure 150 system (Cytiva, Marlborough, MA, USA) (Figure S5b–e). Bound antibodies were eluted with 0.1 M glycine (pH 3.0) and immediately neutralized with 1 M Tris-HCl (pH 9.0). The eluate was dialyzed against storage buffer (20 mM Na_2_HPO_4_, 0.15 M NaCl, pH 7.4) and filtered through a 0.22-µm membrane.

Polishing was performed by anion-exchange chromatography in flow-through mode using Q Sepfast 6HFF resin (Bio-Works Technologies, Uppsala, Sweden) equilibrated with 25 mM Na2HPO4, 0.15 M NaCl (pH 7.5), followed by cation-exchange chromatography in bind-and-elute mode using SP Beads 6FF resin (Smart-Lifesciences, Changzhou, China) equilibrated with 20 mM Tris-HCl (pH 7.5). Elution was performed with a NaCl gradient. The final antibody fractions were dialyzed against storage buffer (20 mM Na_2_HPO_4_, 0.1 M NaCl, pH 7.5), filtered through a 0.22-µm membrane, and stored at 4 °C.

### 4.5. Production and Purification of Recombinant SARS-CoV-2 RBD

Recombinant SARS-CoV-2 RBD Delta was produced by transfecting HEK293T cell (ATCC CRL 3216) cultures with the SBW4G_6xHis_SUMOstar_RBD_Delta genetic construct (Figure S6a). Cell culture supernatant containing recombinant RBD Delta was concentrated by tangential flow filtration using an ÄKTA flux S system (Cytiva, Marlborough, MA, USA) and purified by immobilized metal affinity chromatography (IMAC) on a Ni SepFast column (BioTolomics, Consett, County Durham, UK) using an ÄKTA Pure 150 system (Cytiva, Marlborough, MA, USA). The target protein was eluted by a stepwise increase in imidazole concentration. The N-terminal tag was removed by cleavage with SUMOstar protease (Enzo Life Sciences, Farmingdale, NY, USA), followed by reverse IMAC to separate the tag and protease from the target RBD (Figure S6b). Purified RBD fractions were dialyzed against PBS and stored at 4 °C until use.

### 4.6. Neutralization Assay Against SARS-CoV-2 Variants

All experiments involving authentic SARS-CoV-2 viruses were conducted in a certified BSL-3 facility in strict accordance with biosafety guidelines. Authentic SARS-CoV-2 viruses were propagated in Vero E6 cells (ATCC CRL 1586). Vero E6 cells were seeded in 96-well plates and incubated for 24 h to achieve 90–95% confluency. Recombinant mAbs were serially diluted two-fold in DMEM/F-12 medium (HyClone, Cytiva, MA, USA) and mixed with an equal volume of SARS-CoV-2. After 1 h pre-incubation at 37 °C, the antibody-virus mixtures were added to Vero E6 monolayers in quadruplicate and incubated for 24–72 h at 37 °C. Viral replication was assessed by cytopathic effect (CPE) visualization using gentian violet staining or by immunocytochemistry with mAbs against SARS-CoV-2 (AtaGenix, Beijing, China), followed by detection with Protein G (Abcam, Cambridge, UK) and AEC substrate. Plates were visualized using an EVOS M5000 (Thermo Fisher Scientific, Waltham, MA, USA). The 50% neutralization titer was calculated as the antibody concentration that reduced viral replication by 50% compared to virus-only controls (IC_50_).

### 4.7. ELISA

Inactivated SARS-CoV-2 was used as the antigen. The antigen was diluted in carbonate-bicarbonate buffer and added to 96-well plates (100 µL per well), followed by overnight incubation at 4 °C. Plates were washed 3 times with PBS-T, and non-specific binding sites were blocked with 10 × Fish Gelatin Blocking Agent (Biotium, Fremont, CA, USA) for 2 h at 37 °C. After washing, purified recombinant antibodies (100 ng/mL in PBS-T) were added and incubated for 1 h at 4 °C. Plates were washed 3 times with PBS-T, and immune complexes were detected using Rabbit Anti-Human IgG H&L (HRP) (Abcam, Cambridge, UK) diluted 1:130,000, followed by incubation at 37 °C and 3 washes with PBS-T. As negative controls, cell culture supernatants from CHO-S cells were used. As positive controls, a commercial anti-RBD mAb (1:2000; Sanyou Biopharmaceutical, Shanghai, China) or inactivated serum from a convalescent donor (1:300) was used. TMB substrate (Abcam, Cambridge, UK) was added to the wells and incubated for 15 min. The reaction was stopped with 0.1 M sulfuric acid, and the optical density was measured at 450 nm using a Varioskan LUX microplate reader (Thermo Fisher Scientific, Vantaa, Finland).

### 4.8. Measurement of the Antibody Binding Kinetics with SARS-CoV-2 RBD Delta

The binding kinetics of DEV_K57 and a commercial reference IgG1 (Sanyou Biopharmaceuticals, Shanghai, China) to the SARS-CoV-2 RBD were measured by biolayer interferometry (BLI) using an Octet RED96e instrument (ForteBio, Menlo Park, CA, USA) equipped with anti-penta-HIS biosensors. Following a 2-min baseline step in assay buffer (PBS containing 0.05% Tween 20), the Abs were immobilized onto the biosensors at a concentration of 0.5 µg/mL. The biosensors were then transferred to the assay buffer for a second 1-min baseline, followed by an 8-min association step with the SARS-CoV-2 RBD at a concentration of 5 nM, and a 6-min dissociation step in the assay buffer. The resulting data were analyzed using the Octet Data Analysis software (version 12.0., ForteBio, Menlo Park, CA, USA).

### 4.9. In Vivo Efficacy of DEV_K57 in a Hamster Model

Three groups of male golden Syrian hamsters (*Mesocricetus auratus*, 6–8 weeks old, *n* = 8 per group) were used in this study. All animals were inoculated intranasally with 50 μL of SARS-CoV-2 Delta variant solution containing 50 TCID_50_. The first experimental group received a single intraperitoneal injection of DEV_K57 (5.43 mg/mL, 0.5 mL) 1 h post-infection. The second experimental group received 3 intraperitoneal injections of DEV_K57 (5.43 mg/mL, 0.5 mL each) at 1, 24, and 48 h post-infection. The control group received 3 intraperitoneal injections of 0.9% NaCl (0.5 mL each) at 1, 24, and 48 h post-infection. Viral load in nasal turbinate and lung tissues was determined at 120 h post-infection.

Tissue samples from infected animals were fixed in 10% buffered formalin (BioVitrum, Saint Petersburg, Russia) for 48 h, routinely dehydrated in graded alcohols, cleared in xylene, and embedded in paraffin. Sections (4–5 µm thick) were cut using a Microm HM 360 rotary microtome (Thermo Fisher Scientific, Walldorf, Germany) and stained with hematoxylin and eosin. Light microscopy and microphotography were performed using an Axio Imager Z1 microscope equipped with AxioVision v.4.8.2 software (Carl Zeiss, Jena, Germany). To evaluate the extent and severity of pathological changes, serial tissue sections were digitized using an Olympus VS200 slide scanner (Olympus, Hamburg, Germany).

### 4.10. Statistical Analysis

Statistical analysis was performed using Statistica 13.0 software (TIBCO Software, Palo Alto, CA, USA). Differences between groups were evaluated using non-parametric statistical methods. The Mann-Whitney U test was used for comparisons between 2 groups, and the Kruskal-Wallis test followed by Dunn’s multiple comparison test was applied for comparisons involving more than 2 groups. Statistical significance was defined as a two-sided *p*-value < 0.05.

### 4.11. Preparation of the DEV_K57 Fab-SARS-CoV-2 RBD Delta Complex

The Fab fragment of the DEV_K57 was generated by papain digestion using immobilized papain on a solid-phase support. Digestion products were separated by affinity chromatography on a MabSelect Sure column (Cytiva, Marlborough, MA, USA). The fraction containing Fab fragments was supplemented with the protease inhibitor PMSF (Servicebio, Wuhan, China), concentrated, and stored at −80 °C until further use.

The DEV_K57 Fab-SARS-CoV-2 RBD complexes were formed by mixing the purified recombinant RBD with the DEV_K57 Fab fragment at a molar ratio of 1:1.3. The resulting mixture was incubated at 4 °C for 4 h. The complexes were purified by SEC on a Superdex^®^ 200 Increase 10/300 GL column (GE Healthcare, Chicago, IL, USA) using an ÄKTA Pure 25 system (Cytiva, Marlborough, MA, USA) (Figure S7). A 500-μL aliquot of the incubation mixture was applied to the column and eluted with PBS (pH 7.4) at 4 °C with a flow rate of 0.25 mL/min and a pressure of 0.95 MPa. Fractions containing DEV_K57 Fab-SARS-CoV-2 RBD complex and unbound Fab fragments were collected, analyzed by SDS-PAGE, and concentrated for SEC-SAXS studies.

### 4.12. SEC-SAXS Data Collection and Analysis of the DEV_K57 Fab-SARS-CoV-2 RBD Delta Complex

SEC-SAXS measurement for the DEV_K57 Fab-SARS-CoV-2 RBD Delta complex was performed at the BL19U2 beamline of the Shanghai Synchrotron Radiation Facility (SSRF, Shanghai, China). The X-ray beam size on the stage was 0.33 mm (H) × 0.05 mm (V). A two-dimensional Pilatus3 2M detector (DECTRIS Ltd., Baden, Switzerland) was placed at a sample-to-detector distance of 2.7 m. The scattering vector magnitude range (s = (4π/λ)sinθ, where 2θ is the scattering angle and λ = 0.1033 nm is the wavelength) was 0.07–4.5 nm^−1^.

SEC-SAXS was performed using a Superdex^®^200 Increase 10/300 GL column at 20 °C with a flow rate of 0.5 mL/min. Chromatography was performed on an Agilent 1260 Infinity HPLC system (Agilent Technologies, Santa Clara, CA, USA). The DEV_K57 Fab-SARS-CoV-2 RBD complex sample was loaded at a concentration of 10 mg/mL with an injection volume of 150 μL.

Data collection was performed by continuous acquisition of SAXS frames from the moment of sample injection onto the SEC column until the return to baseline. Primary data processing was carried out using ATSAS v.4.0.1 with CHROMIXS (EMBL, Dublin, Ireland; https://www.embl-hamburg.de/biosaxs/, accessed on 1 July 2026), employed for automated analysis of SEC-SAXS datasets [39,40].

Frame selection was performed by analyzing the SEC elution peak. We evaluated the dependence of the frame-wise radius of gyration (Rg) on the frame index across the range of indices 1084–1517. This dependence was approximated using a Boltzmann sigmoid function. The upper and lower asymptotes were defined by the average Rg values of the early and late regions of the peak, respectively. Each stable region was defined as the range where the modeled Rg value deviated from the corresponding reference asymptote by no more than 20% of the total amplitude. The boundaries of these regions were determined with 95% confidence intervals, yielding ranges of 1119–1209 and 1368–1449. Finally, the UV/dRI ratio analysis was employed to select the final interval for data processing. Frames corresponding to the monodisperse fractions were buffer-subtracted and merged for subsequent analysis.

Guinier analysis, pair-distance distribution function (P(r)) calculation, and ab initio shape reconstruction were performed using PRIMUS, GNOM, and DAMMIF, respectively [41]. The Rg and forward scattering intensity I(0) were determined from Guinier analysis (for sRg < 1.3). MW was estimated using a Bayesian approach.

Ab initio low-resolution shape reconstruction was performed by approximating the molecular envelope with a system of dummy atoms using DAMMIF [42]. The resulting ab initio models were averaged and filtered against the experimental scattering curve using DAMAVER [43]. Superposition of the low-resolution envelope onto the atomic model of the DEV_K57 Fab-SARS-CoV-2 RBD complex was carried out using SUPCOMB [44]. The theoretical scattering curve from the AlphaFold-predicted model was calculated with CRYSOL [45]. Structural models were predicted using AlphaFold3 v3.0.1 via the official AlphaFold Server (Google DeepMind, London, UK; https://alphafoldserver.com/; accessed on 1 July 2026) [46]. The goodness of fit was assessed using the reduced, weighted χ2 statistic. Tertiary structure models were visualized using UCSF ChimeraX v1.15rc (University of California, San Francisco, CA, USA) [47].

## 5. Conclusions

We identified DEV_K57, a broadly neutralizing human mAb that exhibits cross-reactivity, picomolar affinity, and robust in vivo efficacy against SARS-CoV-2. By integrating computational modeling with SEC-SAXS data, we demonstrated that the DEV_K57-RBD Delta binding interface likely differs from previously characterized complex structures in the PDB. These findings underscore the value of rapidly isolating and characterizing antibodies from convalescent donors.

## 6. Patents

1. Shestakova A.A., Dubrovskaya E.V., Prudnikova E.Yu., Imatdinov A.R., Imatdinov I.R., Ivkina D.I. Universal integration vector SB7G_HIGH and recombinant plasmid SB7G_HIGH_DEV_K57, providing synthesis and secretion of recombinant human antibody to receptor-binding domain (RBD) of coronavirus SARS-CoV-2 in mammalian cells and obtained using universal vector SB7G_HIGH, and recombinant monoclonal antibody DEV_K57, having virus-neutralizing activity on SARS-CoV-2. RU2829359C1, 30 October 2024.
2. Ivkina D.I., Shestakova A.A., Prudnikova E.Yu., Dubrovskaya E.V., Imatdinov A.R., Imatdinov I.R. Recombinant plasmid providing for the synthesis and secretion of a recombinant human antibody to the receptor-binding domain (RBD) of the SARS-CoV-2 coronavirus in mammalian cells, and a recombinant monoclonal antibody with virus-neutralising activity against SARS-CoV-2. RU2848470C1, 20 October 2025.
3. Ivkina D.I., Shestakova A.A., Prudnikova E.Yu., Dubrovskaya E.V., Imatdinov A.R., Imatdinov I.R. Recombinant plasmid providing synthesis and secretion of recombinant human antibody to receptor-binding domain (RBD) of SARS-CoV-2 coronavirus in mammalian cells, and recombinant monoclonal antibody having virus-neutralising activity against SARS-CoV-2. RU2856473C1, 24 February 2026.

## Figures and Tables

**Table 1:** Virus-neutralizing activity of mAbs against SARS-CoV-2 Delta and Omicron variants.

№	mAb	Delta		Omicron BA.5.1	
		IC_99_, µg/mL	IC_50_, µg/mL	IC_99_, µg/mL	IC_50_, µg/mL
1	DEV_K57	0.19–1.56	0.006	0.56	0.074
2	DEV_K51	1.56–6.25	0.258	16.00	3.901
3	DEV_K61	0.19–0.39	0.004	30.80	0.441
4	DEV_K55	3.12–12.50	0.748	72.40	0.21
5	DEV_L23	ND	61.25	3.83	1.42
6	DEV_L15	ND	0.80–0.40	ND	ND
7	DEV_L20	1.56	0.78–0.40	ND	ND
8	DEV_K47	3.09	1.54	ND	ND
9	DEV_L38	4.67	2.35	ND	ND
10	DEV_K85	13.20	6.60–1.65	ND	ND
11	DEV_L28	39.84	19.92–9.96	ND	ND
12	DEV_L35	ND	10.00–5.00	ND	ND
13	DEV_K79	ND	15.50–3.88	ND	ND
14	DEV_L45	ND	16.30–8.15	7.65	0.96
15	DEV_K83	ND	13.80	ND	ND
16	DEV_K54	ND	19.80	ND	19.80
17	DEV_L33	ND	25.00–12.50	2.50	1.25
18	DEV_L36	109.50	54.76	ND	21.90
19	DEV_L18	ND	23.20	ND	ND
20	DEV_K50	ND	27.50	ND	ND
21	DEV_K71	ND	27.60	ND	ND
22	DEV_K75	ND	67.50–33.75	ND	ND
23	DEV_K53	ND	151.50–75.75	ND	ND
24	DEV_K87	ND	133.75	ND	ND
25	DEV_K77	ND	ND	ND	6.05
26	DEV_K66	ND	ND	14.18	7.09
27	DEV_L17	ND	ND	ND	12.00
Other mAbs (11) *		ND	ND	ND	ND

Note: ND—virus-neutralizing activity was not detected. An mAb was considered to have virus-neutralizing activity if IC_50_ < 15.00 µg/mL. * Other mAbs (11): DEV_L30, DEV_L32, DEV_L3, DEV_K52, DEV_L22, DEV_L34, DEV_L41, DEV_L42, DEV_K46, DEV_K49, and DEV_K69.

**Table 2:** Virus-neutralizing activity of DEV_K57 against SARS-CoV-2 variants.

SARS-CoV-2 Variant	GISAID ID	IC_50_, µg/mL
Wuhan	EPI_ISL_406844	0.005
Alpha	EPI_ISL_6565012	0.0005
Beta	EPI_ISL_6492245	0.0005
Gamma	EPI_ISL_6565014	0.0005
Delta	EPI_ISL_7338814	0.0005
Omicron BA.1	EPI_ISL_8920444	0.005
Omicron BA.2	EPI_ISL_12809000	0.005
Omicron CH.1.1	EPI_ISL_17730071	50
Omicron BN.1.3	EPI_ISL_17678725	0.0005
Omicron CL.1.2	EPI_ISL_17678727	15.63
Omicron BA.5.1	EPI_ISL_16613435	7.81
Omicron BA.5.2	EPI_ISL_16613436	5.0
Omicron BQ.1.1	EPI_ISL_17730076	5.0
Omicron XBB.1.5	EPI_ISL_17730074	250
Omicron XBB.3	EPI_ISL_16520275	500
Omicron EG.5.1	EPI_ISL_18520144	>500
Omicron XBB.1.16	EPI_ISL_18520146	125
Omicron JN.1	EPI_ISL_19035845	>500

**Table 3:** Oligonucleotides used for the production of immunoglobulin heavy and light chain genes.

Oligonucleotide Name	Oligonucleotide Sequences
R_hIGH_1	CTT GTC CAC CTT GGT GTT GCT GGG CTT GTG
R-hIGK_1	GTT TCT CGT AGT CTG CTT TGC TCA GCG TCA G
R-hIGL_1	GAC TTC CAC TGC TCR GGC GTC AGG CTC A
R_hIGH_3	GAA GTA GTC CTT GAC CAG GCA GCC CAG
R-hIGK_3	TCT GGG ATA GAA GTT ATT CAG CAG GCA CAC
R-hIGL_3	TCA GAG GAG GGT GGG AAC AGA GTG AC
F-iL_B	ACG TGC TGG TTA TTG TGC TGT CTC ATT GGC TCG AGT TTT TCA GCA AGA
F-iRACE-primer_C	ATG GCT CGA GTT TTT CAG CAA GAT

## Data Availability

The SEC-SAXS dataset supporting this study has been deposited in the Zenodo repository and is publicly available at: https://doi.org/10.5281/zenodo.22718714. Further inquiries can be directed to the corresponding author.

## Supplementary Materials

The following supporting information can be downloaded at: https://www.mdpi.com/article/10.3390/ijms27219445/s1.

## Abbreviations

The following abbreviations are used in this manuscript:

6xHis	hexahistidine tag
5’-TSO	5’-template switching oligonucleotide
ACE2	angiotensin-converting enzyme 2
AEC	3-amino-9-ethylcarbazole
ATCC CRL	american type culture collection cell line
ATSAS	automated toolbox for small-angle scattering data analysis
a.u.	arbitrary units
bp	base pairs
BLI	biolayer interferometry
CD19	cluster of differentiation 19
cDNA	complementary deoxyribonucleic acid
CH/CL	constant heavy/constant light
CHO-S	chinese hamster ovary suspension
CO_2_	carbon dioxide
COVID-19	coronavirus disease 2019
CPE	cytopathic effect
Dmax	maximum dimension
DMEM/F-12	Dulbecco’s modified eagle medium F-12
DNA	deoxyribonucleic acid
E	envelope protein
ELISA	enzyme-linked immunosorbent assay
Fab	fragment antigen-binding
Fc	fragment crystallizable
FSC-A	forward scatter area
FSC-H	forward scatter height
FVS620	fixable viability stain 620
GISAID	Global Initiative on Sharing All Influenza Data
HEK293T	human embryonic kidney 293 cells expressing the large T antigen
HPLC	high performance liquid chromatography
HRP	horseradish peroxidase
h	hours
I(0)	forward scattering intensity
IC_50_	50% inhibitory concentration
ID	identifier
IgG	immunoglobulin g
IGHV	immunoglobulin heavy chain variable region
IGKV	immunoglobulin kappa chain variable region
IGLV	immunoglobulin lambda chain variable region
IMAC	immobilized metal affinity chromatography
I(s)	scattering intensity as a function of scattering vector
K_D_	dissociation constant
kDa	kilodalton
M	membrane protein
mAb/mAbs	monoclonal antibody / antibodies
MW	molecular weight
N	nucleocapsid protein
Na_2_HPO_4_	disodium phosphate
NaCl	sodium chloride
ND	not detected
nm	nanometer
nM	nanomolar
ns	not significant
PBS	phosphate-buffered saline
PBS-T	phosphate-buffered saline with tween
PCR	polymerase chain reaction
PDB	protein data bank
PerCP-Cy5.5	peridinin chlorophyll protein—cyanine 5.5
pH	potential of hydrogen
pM	picomolar
PMSF	phenylmethylsulfonyl fluoride
P(r)	pair-distance distribution function
RACE	rapid amplification of cdna ends
RBD	receptor binding domain
RBM	receptor-binding motif
RNA	ribonucleic acid
RT	reverse transcription
S	spike glycoprotein
SARS-CoV-2	severe acute respiratory syndrome coronavirus 2
SDS-PAGE	sodium dodecyl sulfate—polyacrylamide gel electrophoresis
SEC-SAXS	size-exclusion chromatography coupled with small angle x-ray scattering
SSC-A	side scatter area
SSRF	shanghai synchrotron radiation facility
ssRNA(+)	positive-sense single-stranded ribonucleic acid
SUMO	small ubiquitin-like modifier
TCID_50_	50% tissue culture infectious dose
TMB	3,3’,5,5’-tetramethylbenzidine
VL/VH	variable light/ variable heavy
UCSF	University of California, San Francisco
