# Supplementary material for: The Broadly Neutralizing DEV_K57 Antibody, Isolated from a Convalescent Patient, in Complex with the SARS-CoV-2 RBD
Source: Int J Mol Sci. 2026 Oct 23;27(21):9445. doi: 10.3390/ijms27219445 (PMC13762534; doi:10.3390/ijms27219445)
Supplement: Supplementary file 1 [file ijms-27-09445-s001.zip › ijms-4619916-supplementary.pdf]

The broadly neutralizing DEV_K57 antibody, isolated from a convalescent patient, in complex with the SARS-CoV-2 RBD

Elina V. Sinitsina[1], Ilnaz R. Imatdinov[1], Vitaliy Y. Tuyrin[1], Andrey V. Shipovalov[1], Anna V. Zaykovskaya[1], Irina A. Osinkina[1], Alexey O. Yanshin[1], Nikita S. Radchenko[1], Yury L. Ryzhykau[2], Vsevolod V. Sudarev[2], Daria D. Kuklina[2], Galina S. Onkhonova[1], Elena K. Ivleva[1], Alexander P. Agafonov[1], and Anastasia V. Gladysheva[1,*]

[1]State Research Center of Virology and Biotechnology "Vector", 630559 Kol'tsovo, Russia
[2]Moscow Center for Advanced Studies, 123592 Moscow, Russia
*****Correspondence: gladysheva_av@vector.nsc.ru

SUPPLEMENTARY MATERIAL

Table S1. ELISA results of mAbs against inactivated SARS-CoV-2 Delta and Omicron BA.5.1 variants.

mAb	Optical density (450 nm)		mAb	Optical density (450 nm)	
	Delta	Omicron		Delta	Omicron
DEV_K57	0.7297	2.6565	DEV_K83	0.1811	1.5295
DEV_K51	0.3392	1.3994	DEV_L33	0.1369	2.3044
DEV_K61	1.1091	2.3997	DEV_L36	0.8472	1.9400
DEV_K55	1.7854	2.6765	DEV_L18	0.1332	0.8665
DEV_L23	0.1881	1.9479	DEV_L30	0.5981	1.2449
DEV_L15	0.5642	1.3301	DEV_K71	0.1699	0.8084
DEV_L20	0.8866	0.7222	DEV_K75	0.4681	1.6098
DEV_K47	0.5626	1.4178	DEV_K53	1.5478	1.9777
DEV_L38	1.2766	0.3088	DEV_K77	0.8454	2.0075
DEV_K85	1.0114	0.3246	DEV_K66	0.1759	2.6997
DEV_L28	1.7857	0.9945	DEV_L17	0.2428	2.5026
DEV_L03	0.5547	2.1761	DEV_K87	0.1939	2.0173
DEV_K79	0.1674	1.4397	DEV_K54	0.3711	0.6481
DEV_L45	0.1808	2.1851	DEV_L32	0.7826	1.7078
DEV_L41	0.1753	2.4316	DEV_K46	0.2125	1.4464
DEV_K69	0.2799	1.5472	DEV_L34	0.1209	2.2556
DEV_K49	0.2489	0.2731	DEV_L42	0.1908	0.4576
DEV_L35	0.3870	0.3873	DEV_K50	0.2014	0.6284
DEV_L22	0.1287	0.2827	DEV_K52	0.2296	0.3989
K+	4.7104	4.4601	K-	0.1124	0.2561

Note: An mAb was considered reactive if OD450>0.33 for the Delta variant and OD450>0.75 for the Omicron variant.

In the table, mAbs that bound both the Delta and Omicron variants in the ELISA are highlighted in green; antibodies that bound the Delta variant only are highlighted in yellow; antibodies that bound the Omicron variant only are highlighted in orange; antibodies that showed no specific binding to either variant are highlighted in red.

Table S2. Comparison of ELISA and neutralization assay results against SARS-CoV-2 Delta and Omicron BA.5.1 variants for 38 mAbs.

№	mAb	ELISA		Neutralization	
		Delta	Omicron	Delta	Omicron
1	DEV_K57	+	+	+	+
2	DEV_K51	+	+	+	+
3	DEV_K61	+	+	+	+
4	DEV_K55	+	+	+	+
5	DEV_L23	-	+	-	+
6	DEV_L15	+	+	+	-
7	DEV_L20	+	-	+	-
8	DEV_K47	+	+	+	-
9	DEV_L38	+	-	+	-
10	DEV_K85	+	-	+	-
11	DEV_L28	+	+	-	-
12	DEV_L03	+	+	-	-
13	DEV_L35	+	-	+	-
14	DEV_K79	-	+	+	-
15	DEV_L45	-	+	+	+
16	DEV_K83	-	+	+	-
17	DEV_L41	-	+	-	-
18	DEV_K69	-	+	-	-
19	DEV_K54	+	-	-	-
20	DEV_L33	-	+	+	+
21	DEV_L36	+	+	-	-
22	DEV_L18	-	+	-	-
23	DEV_K50	-	-	-	-
24	DEV_K71	-	+	-	-
25	DEV_L30	+	+	-	-
26	DEV_K75	+	+	-	-
27	DEV_K53	+	+	-	-
28	DEV_K87	-	+	-	-
29	DEV_K77	+	+	-	+
30	DEV_K66	-	+	-	+
31	DEV_L17	-	+	-	+
32	DEV_L32	+	+	-	-
33	DEV_L34	-	+	-	-
34	DEV_K46	-	+	-	-
35	DEV_K52	-	-	-	-
36	DEV_K49	-	-	-	-
37	DEV_L22	-	-	-	-
38	DEV_L42	-	-	-	-

Note: Antibodies that possess neutralizing activity against the Delta and Omicron variants and are able to specifically interact with the RBD of both virus variants are highlighted in green; ELISA results for antibodies capable of specifically interacting with the RBD of both virus variants are highlighted in orange; results of the virus neutralization assay for antibodies that possess activity against the Delta and Omicron variants are highlighted in blue.

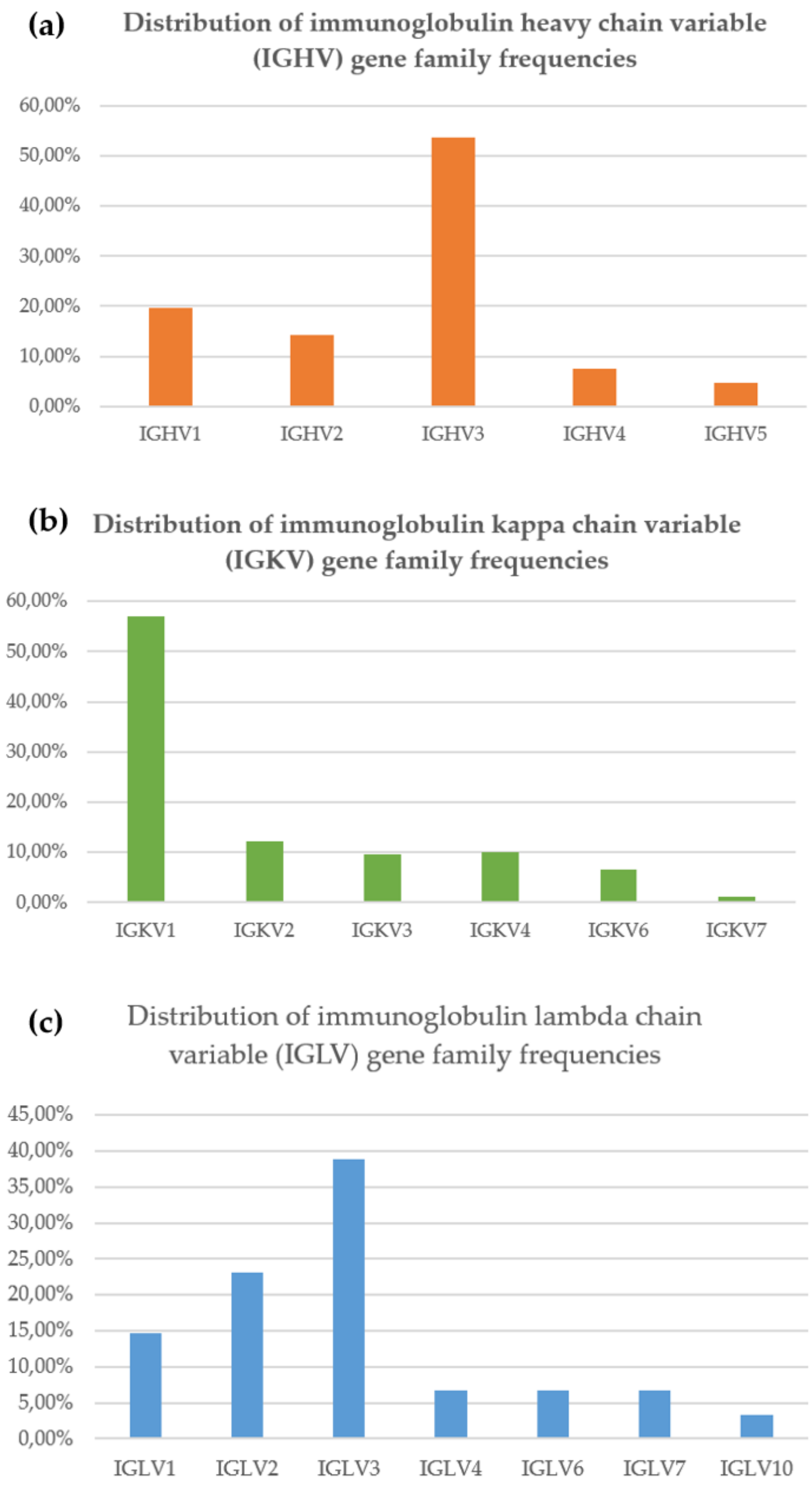

Figure S1. Distribution of immunoglobulin variable gene family frequencies ((a) IGHV, (b) IGKV, and (c) IGLV) in the analyzed repertoire.

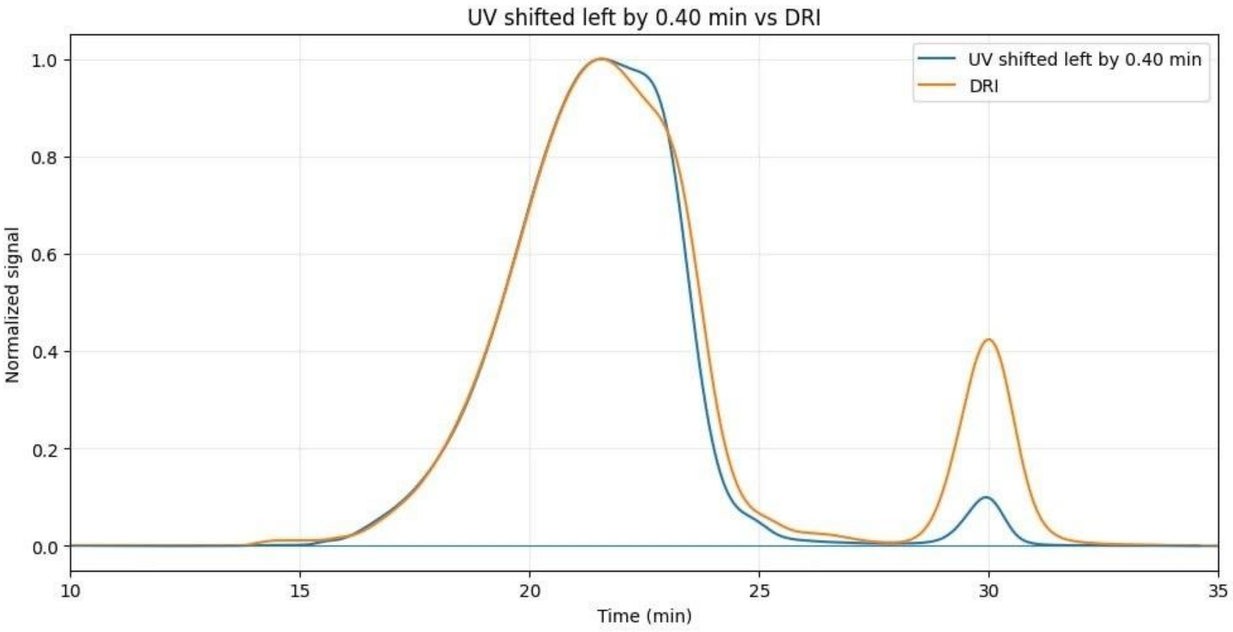

Figure S2. UV/dRI ratio profile during size-exclusion chromatography.

Table S3. Foldseek results for the DEV_K57 (AlphaFold 3 model) against the different mAbs from PDB**.**

Entry	Description	Chain	RMSD	TM-score	Seq. Identity	Aligned Residues	Seq. Length
AF3 model	AlphaFold 3 model of the DEV_K57 Fab-SARS-CoV-2 RBD Delta complex	A	-	-	-	-	239
8DNN	Crystal structure of neutralizing antibody 80 in complex with SARS-CoV-2 receptor binding domain	E	1.68	0.89	87%	218	227
8BG1	Crystal structure of the SARS-CoV-2 S RBD in complex with pT1511 scFV	D	1.60	0.89	73%	217	237
8C3V	SARS-CoV-2 Delta-RBD complexed with BA.2-13 Fab and C1 nanobody	A	1.65	0.88	70%	213	231
7XSC	Crystal structure of SARS-CoV-2 spike receptor binding domain bound with P5S-2B10	A	2.14	0.78	74%	195	220
8DCC	SARS-CoV-2 Receptor-Binding Domain SPEEDesign Immunogen 3 Bound to P2B-2F6 Fab	C	2.10	0.87	69%	216	242

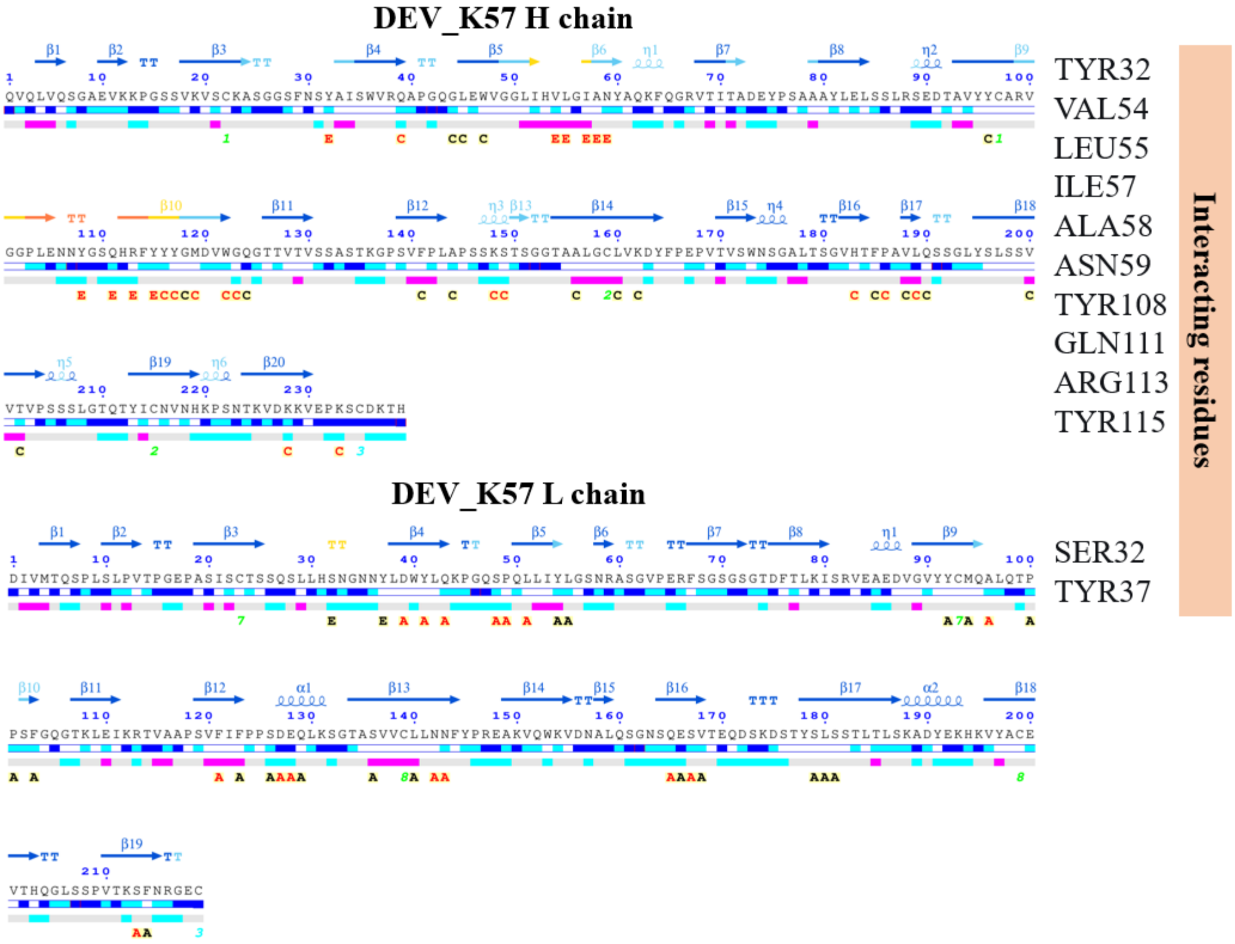

Figure S3. Amino acid sequence analysis of the DEV_K57 heavy (H) and light (L) chains. The left panel displays the primary and secondary structures of the DEV_K57 H and L chains. Secondary structure is color-coded according to pLDDT, with blue indicating very high confidence and yellow indicating low confidence. The relative accessibility calculated by DSSP for each residue is shown with a colored bar below the sequences block: white is buried, cyan is intermediate, and blue is accessible. The hydropathy calculated from the query sequence using the Kyte and Doolittle algorithm is shown by a second colored bar below the accessibility: pink is hydrophobic, grey is intermediate, and cyan is hydrophilic. The right panel highlights the specific amino acid residues of the DEV_K57 involved in the interaction with the RBD.

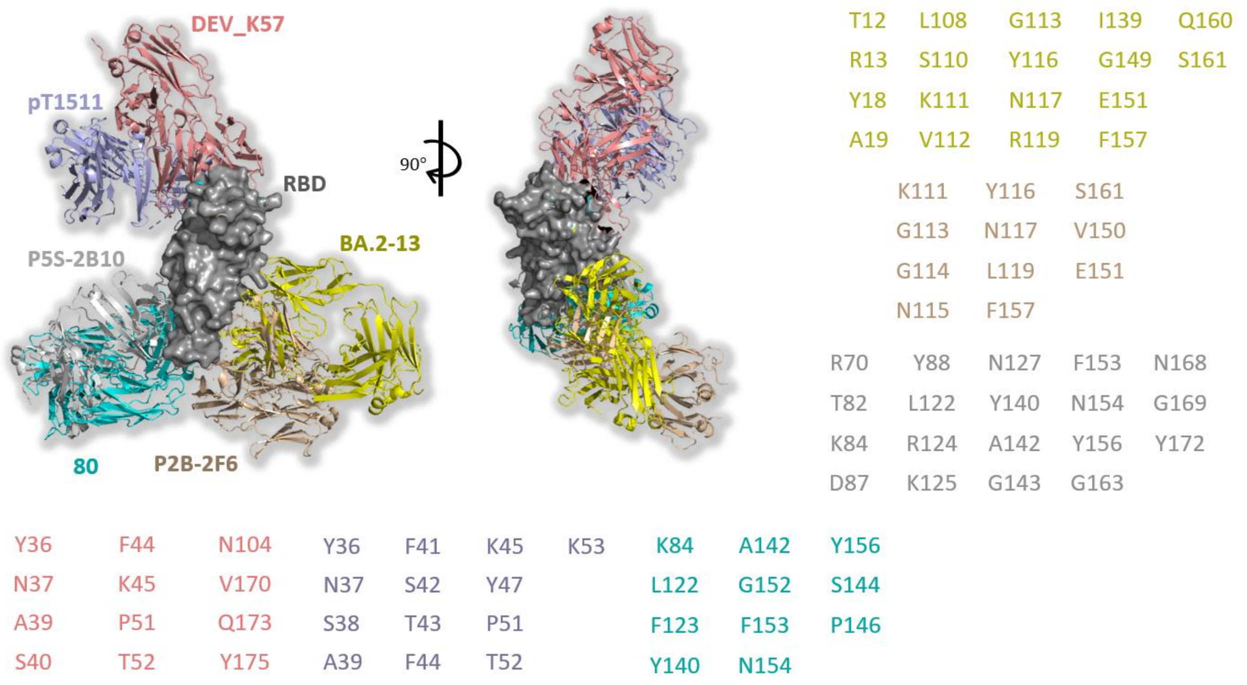

Figure S4. Structural superposition of SARS-CoV-2 RBD-mAb complexes. The complexes shown are DEV_K57 (pink), pT1511 (blue; PDB ID: 8BG1), P5S-2B10 (gray; PDB ID: 7XCS), 80 (cyan; PDB ID: 8DNN), P2B-2F6 (brown; PDB ID: 8DCC), and BA.2-13 (yellow; PDB ID: 8C3V). The SARS-CoV-2 RBD is depicted as a gray molecular surface, while the mAbs are shown in ribbon representation. The lower panel provides a detailed view of the interaction interfaces between the RBD and mAbs. The color of the amino acid residues corresponds to the respective antibody.

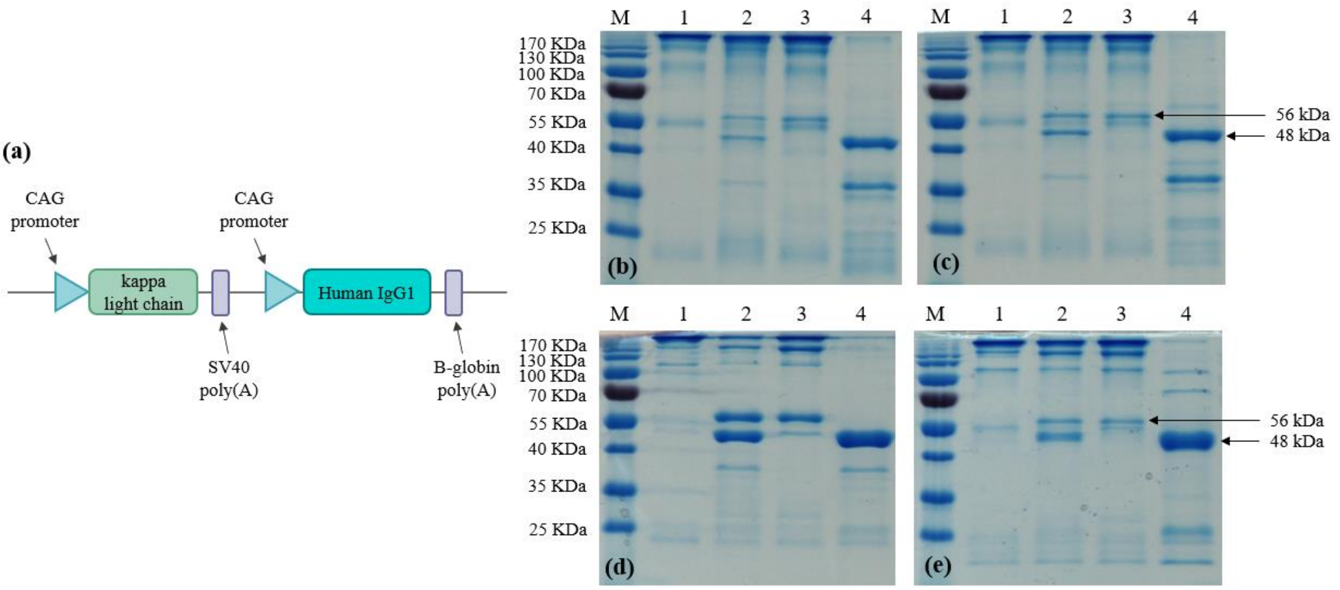

Figure S5. Schematic design of the pCAG_mAb expression cassette used for recombinant antibody production in CHO cells (A), and SDS-PAGE analysis of recombinant antibody samples: DEV_K51 (B), DEV_K55 (C), DEV_K57 (D), and DEV_K61 (E). Lanes: 1, intact full-length monoclonal antibody; 2, crude papain digestion products; 3, fraction containing undigested antibody and Fc fragments eluted from Protein A affinity chromatography; 4, purified Fab fragments collected in the flow-through during Protein A affinity chromatography; M, PageRuler™ Prestained Protein Ladder molecular weight marker (Thermo Fisher Scientific, Waltham, MA, USA).

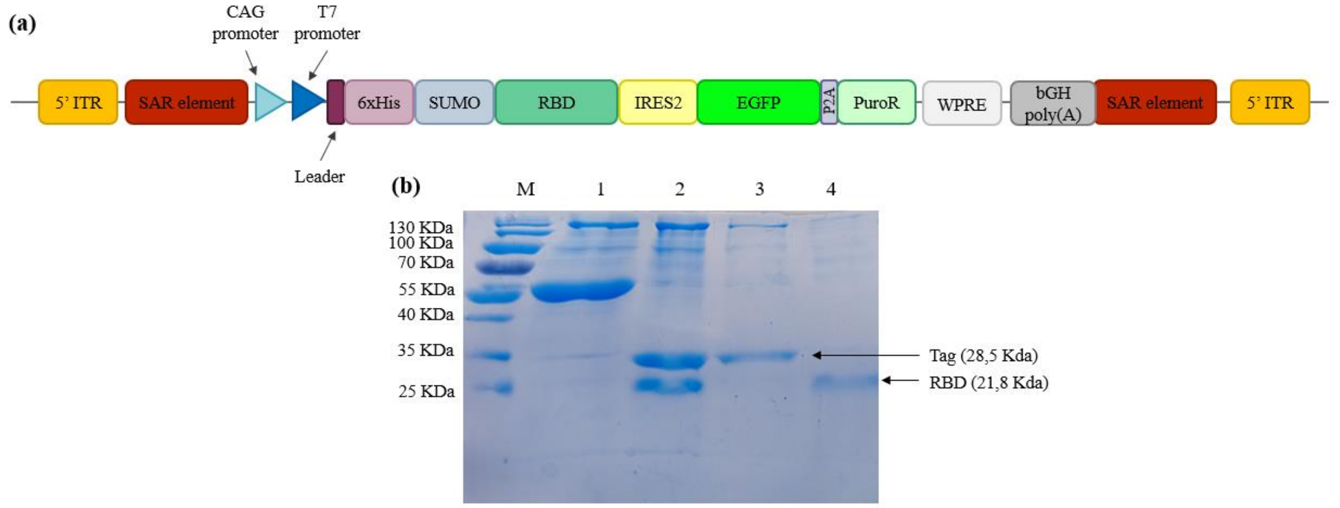

Figure S6. Schematic design of the SBW4G_6xHis_SUMOstar_RBD_Delta expression cassette used for recombinant SARS-CoV-2 RBD Delta production in HEK293T cells (a), and SDS-PAGE analysis of the purification process (b). Lanes: 1, chimeric fusion protein comprising the recombinant RBD and N-terminal tags; 2, cleavage products of the fusion protein following digestion with SUMOstar protease; 3, cleaved N-terminal tag fraction separated by affinity chromatography; 4, purified recombinant SARS-CoV-2 RBD Delta obtained after tag removal via affinity chromatography; M, PageRuler™ Prestained Protein Ladder molecular weight marker (Thermo Fisher Scientific, Waltham, MA, USA).

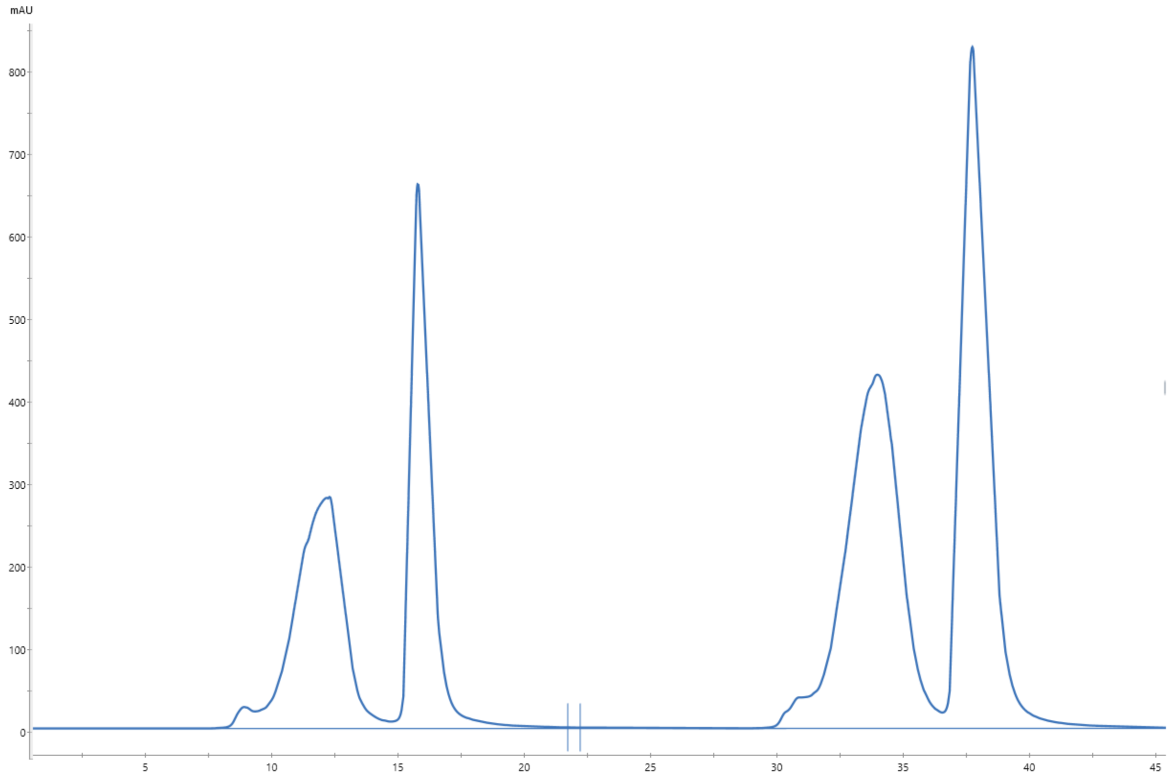

Figure S7. Size-exclusion chromatography elution profile. The chromatogram displays four peaks resulting from two consecutive injections of the sample. For each injection, the first peak corresponds to the DEV_K57 Fab-SARS-CoV-2 RBD Delta complex, and the second peak represents the unbound (free) DEV_K57 Fab.
